# Is Femoral Head Bone Marrow Edema of Unknown Etiology Associated with Acetabular Overcoverage? A CT-Based Three-Dimensional Study

**DOI:** 10.3390/tomography12020019

**Published:** 2026-02-04

**Authors:** Veli Süha Öztürk, Tubanur Şanlı, Ali Balcı, Onur Hapa

**Affiliations:** 1 Department of Radiology, Faculty of Medicine, Ondokuz Mayıs University, Samsun 55200, Turkey; 2 Department of Radiology, Faculty of Medicine, Dokuz Eylül University, Izmir 35330, Turkey; 3 Department of Orthopedics and Traumatology, Faculty of Medicine, Dokuz Eylül University, Izmir 35330, Turkey

**Keywords:** femoral head bone marrow edema, femoroacetabular impingement, pincer type impingement, computed tomography maximum intensity projection, acetabular overcoverage

## Abstract

**Background:** This study aimed to investigate the association between femoroacetabular impingement (FAI) morphology and femoral head bone marrow edema of unknown etiology on hip magnetic resonance imaging (MRI), and to assess the added value of computed tomography-based three-dimensional maximum intensity projection (CT-MIP) measurements in identifying a predisposition to acetabular overcoverage. **Methods:** Hip MRI examinations performed between January 2007 and 2025 were retrospectively reviewed. Cases with bone marrow edema attributable to identifiable etiologies were excluded. Twenty-six patients with available hip or pelvis computed tomography (CT) examinations obtained within one year were included, along with an age- and sex-matched control group imaged for indications unrelated to hip pain. A total of 104 hip joints were evaluated. Alpha angles were measured on axial oblique CT reformations. Virtual pelvic radiographs generated from CT-based three-dimensional reconstructions were used for lateral center-edge angle (LCEA) measurements, and acetabular coverage was quantified using the acetabular coverage index derived from CT-MIP images. Appropriate statistical analyses were performed, with *p* < 0.05 considered statistically significant. **Results:** FAI was identified in 82.7% of cases with bone marrow edema of unknown etiology on MRI (*p* < 0.001), with pincer-type morphology being the most prevalent subtype (55.8%). Bone marrow edema was significantly more common in pincer-type FAI compared with other subtypes (*p* < 0.001) and predominantly involved the posterolateral femoral head. Mean alpha angle, LCEA, and acetabular coverage index values were significantly higher in the case group than in controls (*p* < 0.001). For the detection of pincer-type FAI, CT-MIP-based acetabular coverage index demonstrated superior diagnostic performance compared with LCEA (AUC, 0.917 vs. 0.855; *p* = 0.017), with an optimal cutoff value of 0.93 yielding high specificity and accuracy. All measurements showed excellent intraobserver and interobserver reliability. **Conclusions:** Femoral head bone marrow edema of unknown etiology may serve as a radiologic clue to underlying pincer-type FAI, while CT-MIP-based analyses may provide incremental value beyond conventional angular measurements in characterizing acetabular overcoverage.

## Simple Summary

Atypical focal bone marrow edema of unknown etiology in the femoral head on hip magnetic resonance imaging can be difficult to interpret in daily practice. Our findings suggest that this imaging pattern is frequently associated with pincer-type femoroacetabular impingement. Three-dimensional computed tomography-based measurements, particularly the acetabular coverage index, improved the identification of this morphology compared with conventional angular measurements. Recognizing this association may assist radiologists in detecting an underlying structural cause of edema, support clinical decision-making, and encourage further research to expand these observations in larger and more diverse patient populations.

## 1. Introduction

On magnetic resonance imaging (MRI) performed for hip pain, femoral head T2-weighted bone marrow edema-like signal hyperintensity reflects a broad differential diagnosis, ranging from avascular necrosis and transient osteoporosis to stress reaction and osteomyelitis [1,2,3]. While imaging findings allow identification of the underlying etiology of bone marrow edema in the majority of cases, a subset of patients exhibits focal bone marrow edema with atypical distribution and morphology that does not fully correlate with clinical symptoms. This underrecognized entity represents a diagnostically challenging subgroup in routine clinical practice. Despite limited detailed characterization in the literature, focal bone marrow edema occurring in characteristic locations and patterns is frequently considered by experienced radiologists and orthopedic surgeons to represent a manifestation associated with femoroacetabular impingement (FAI) [4]. However, in the absence of robust supporting evidence, this clinical observation has largely remained an imaging finding whose diagnostic significance is not yet well established in routine reporting.

FAI is a mechanically driven condition caused by abnormal contact between the femoral head–neck junction and the acetabulum during hip motion and most commonly affects young to middle-aged adults between 30 and 50 years of age [5,6]. Notably, this age range also corresponds to the primary target population of the increasingly emphasized concept of “well-aging”. During this critical period, in which musculoskeletal “biological capital” is accumulated, gains or losses in bone and muscle health may directly influence the trajectory of aging after the fourth decade of life and ultimately determine long-term joint health [7,8]. At the core of this concept lies the muscle-bone-adipose tissue triad, within which FAI may initiate a self-perpetuating cycle by inducing pain and limiting joint motion, thereby adversely affecting all three components. Joint pain and functional impairment lead to reduced physical activity, resulting over time in atrophy of the gluteal and quadriceps muscles and subsequent development of sarcopenia. Progressive sarcopenia compromises joint stability, while a concomitant reduction in basal metabolic rate promotes weight gain. In turn, increased body weight and muscle weakness may accelerate FAI-related chondral and labral damage, ultimately contributing to the progression of osteoarthritis (OA) [9]. As OA progresses, mobility becomes further restricted and pain intensifies, leading to increasingly sedentary behavior and the emergence of a broader vicious cycle associated with comorbidities such as hypertension, diabetes mellitus, obesity, and depression. Consequently, focal and atypical femoral head bone marrow edema, initially perceived as a seemingly innocuous imaging finding, should be carefully considered if it represents a potentially relevant imaging finding within this pathological cascade, warranting heightened clinical attention and awareness.

Although three-dimensional computed tomography (3D CT) and MRI-based techniques have been increasingly used to evaluate femoroacetabular morphology and acetabular coverage, the imaging significance of focal femoral head bone marrow edema of unknown etiology within this context remains incompletely characterized. In particular, its relationship with pincer-type acetabular overcoverage has not been systematically examined using practical CT-based approaches applicable to routine clinical imaging. While conventional radiography remains the first-line modality for the assessment of FAI, its two-dimensional nature and sensitivity to pelvic positioning limit accurate depiction of complex acetabular morphology. In contrast, CT-based reconstructions allow a more comprehensive evaluation of acetabular coverage, particularly in cases of subtle or region-specific pincer-type overcoverage [10,11,12].

Based on these considerations, we sought to evaluate the association between femoral head bone marrow edema of unknown etiology and FAI. In cases with bone marrow edema unexplained by another pathology, FAI assessment incorporated computed tomography based 3D maximum intensity projection (CT-MIP) reconstructions in addition to conventional radiographic evaluation to allow detailed morphological analysis of the acetabular rim.

## 2. Materials and Methods

### 2.1. Study Design

**Figure 1:**
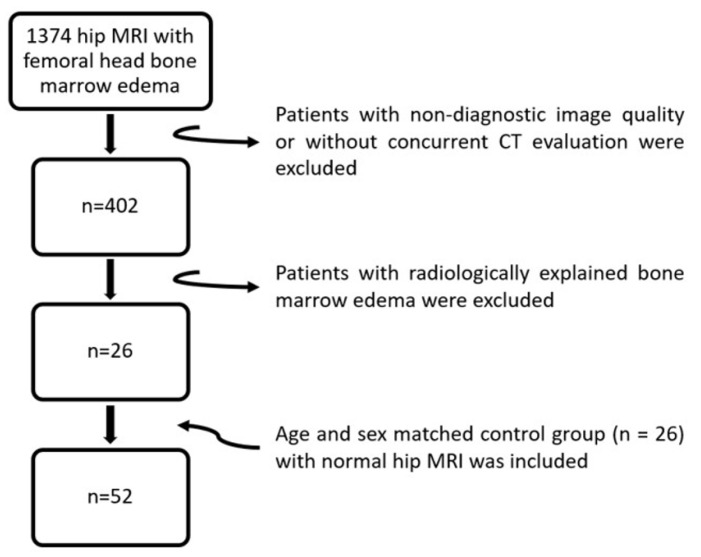
Flowchart of patient selection.

We retrospectively reviewed hip MRI examinations performed between January 2007 and January 2025. All MRI examinations were independently reviewed by two radiologists for the presence of focal atypical bone marrow edema within the femoral head. Atypical bone marrow edema was specifically sought when confined to the femoral head or head–neck junction, measuring no more than 2 cm in greatest dimension, predominantly subcortical in distribution, not involving the entire femoral head, and not attributable to an alternative identifiable cause. In cases of discrepant interpretation, disagreements were resolved by consensus. Eligibility criteria included patients aged 18–75 years with available hip radiography and at least one computed tomography (CT) examination of the hip or pelvis performed within one year of the index MRI. Cases with bone marrow edema attributable to identifiable causes—including avascular necrosis, transient osteoporosis, complex regional pain syndrome, trauma, infectious or inflammatory arthritis, metabolic arthropathy, or advanced-stage osteoarthritis—were excluded. Additional exclusion criteria included malignancy, ligamentous injury, radiologically suspected labral tears, prior hip surgery, hip dysplasia, subchondral insufficiency fractures, osteochondral lesions, as well as MRI examinations of insufficient diagnostic quality due to artifacts or suboptimal imaging technique (Figure 1). To ensure consistency and reduce subjectivity, the exclusion of alternative identifiable causes was performed using a structured review process. All MRI examinations were systematically evaluated for imaging findings corresponding to the predefined exclusion diagnoses listed above. In addition, available clinical records at the time of imaging were reviewed to identify relevant trauma history, systemic disease, or inflammatory or infectious conditions. Where available, radiographic and CT examinations were used for imaging correlation. Image interpretation was performed independently by two radiologists, and cases with discrepant assessments were resolved by consensus. Ultimately, 26 patients were included in the case group. An equal number of individuals comprised the control group, which was selected from the same institutional imaging database as the case group. For each case, one age- and sex-matched control hip was identified using individual matching, with age matched within a ±2-year range and exact sex matching. When multiple eligible control candidates were available, selection was performed randomly. Control subjects had no history of hip pain, trauma, surgery, or known hip pathology. The same exclusion criteria applied to the case group were also applied to the control group. In total, 104 hip joints were evaluated. This retrospective, single-center study was approved by the institutional ethics committee, and the requirement for informed consent was waived in accordance with guidelines for retrospective analyses.

**Figure 2:**
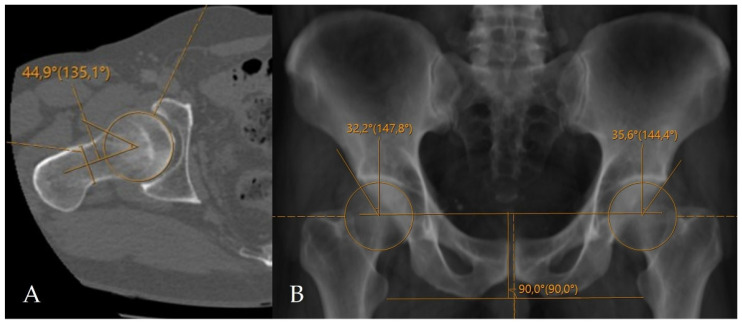
Application of conventional angular measurement methods on a radiology workstation. (**A**) Measurement of the alpha angle on axial oblique CT reformats. (**B**) Measurement of the LCEA on a CT-based virtual pelvic radiograph.

**Figure 3:**
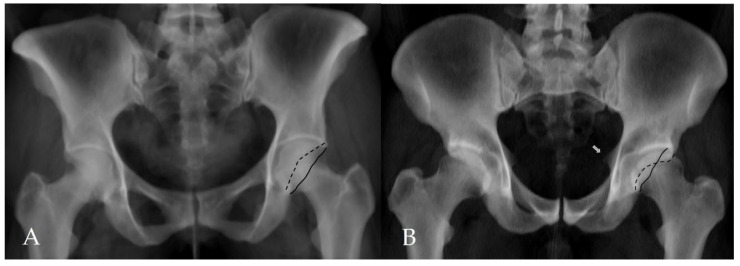
Assessment of additional morphological parameters of femoroacetabular impingement on a CT-based virtual pelvic radiograph. (**A**) Negative acetabular crossover sign. The black solid line represents the posterior acetabular wall, and the black dashed line represents the anterior acetabular wall. (**B**) Positive acetabular crossover sign with an associated ischial spine sign (white arrow), suggestive of pincer-type femoroacetabular impingement and acetabular retroversion.

The alpha angle and lateral center–edge angle (LCEA) were measured on CT. The alpha angle was assessed on axial oblique CT reformats parallel to the femoral neck axis as described by Nötzli et al., and the LCEA was measured using the CT-based coronal three-dimensional (3D) virtual pelvis radiograph model as used by Ergen et al. [13,14]. Acetabular coverage index (ACI) values were subsequently calculated using CT-MIP reconstructions. In all cases, angular measurements were complemented by a comprehensive morphological assessment for the presence of FAI, including evaluation of the crossover sign, posterior wall sign, ischial spine sign, and anterior wall lateralization (Figure 2 and Figure 3). CT was further used to evaluate the morphology of the acetabular rim, and acetabular overcoverage subtypes were determined [15]. The presence of underlying FAI and its specific subtype were determined by two radiologists in consensus. All measurements were independently repeated by both radiologists at a four-week interval. The senior radiologist had 20 years of musculoskeletal radiology experience, whereas the general radiologist had 5 years of clinical radiology experience. Prior to the measurements, the general radiologist underwent a one-week training period under the supervision of the senior radiologist. Statistical analyses were performed using the measurements obtained by the senior musculoskeletal radiologist. To assess intraobserver reliability, the senior radiologist recalculated ACI measurements after a minimum interval of two months. Prior to initiating angular measurements, the entire dataset was reviewed and verified by an independent radiology resident after deletion of all previous measurements and anonymization of cases. Subsequently, cases were randomly ordered to minimize assessment bias and ensure objective analysis. For primary statistical analyses, measurements obtained by the senior musculoskeletal radiologist were used to reflect routine clinical practice, while the second reader’s measurements were used exclusively for interobserver agreement analysis.

### 2.2. Imaging Protocols and Interpretation of Angular and Acetabular Coverage Measurements

Computed tomography (CT) images were obtained in the radiology department of our institution using 16-, 64-, and 128–detector-row multislice CT scanners (Brilliance 16, Brilliance 64, and Ingenuity; Philips Medical Systems B.V., Best, The Netherlands). For CT examinations, the following parameters were used: a tube voltage of 120 kV, a slice thickness of 1 mm, and a 512 × 512 matrix. The tube current–time product (mAs) was adjusted according to patient body mass index. All magnetic resonance (MR) images were acquired using two 1.5-T Philips MRI systems (Achieva and Ingenia; Philips Medical Systems B.V., The Netherlands). All examinations were performed using either a 6-channel body coil or an 8-channel body flex coil. Standard hip MRI sequence parameters are summarized in Appendix A, Table A1.

**Figure 4:**
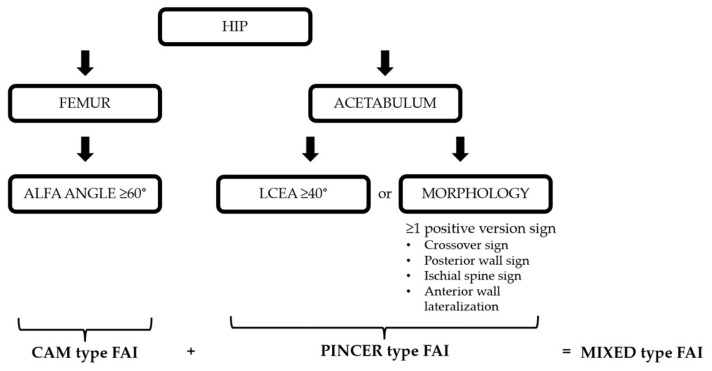
Pre-specified diagnostic algorithm for femoroacetabular impingement classification.

**Figure 5:**
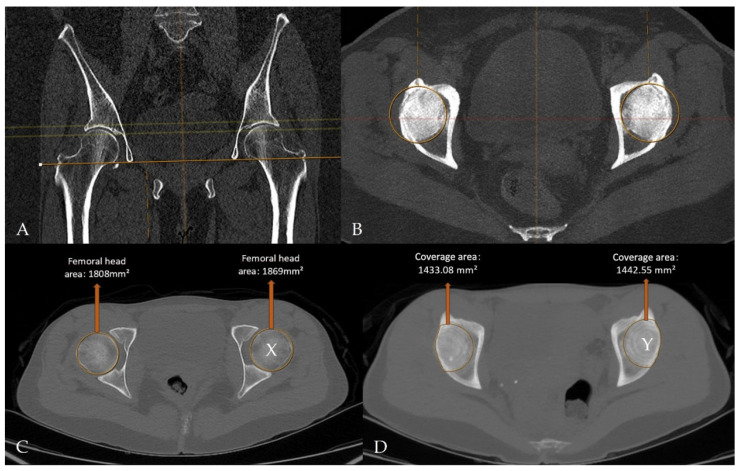
CT- and MIP-derived images illustrate quantitative assessment of femoral head morphology and acetabular coverage on a radiology workstation. (**A**,**B**) Axial MIP reformats aligned using the teardrop landmarks, demonstrating CT-MIP processing on the imaging workstation. (**C**) Maximal femoral head cross-sectional area measured on axial CT images. (**D**) Acetabular coverage area segmented on multiplanar MIP reconstructions. The acetabular coverage index was calculated as Y/X (right, 0.792632; left, 0.771829).

Imaging data were retrospectively retrieved from the SECTRA PACS workstation (IDS7, version 24.2.16.6066; Sectra AB, Linköping, Sweden). Cam-type FAI was defined by an alpha angle of ≥60°, whereas pincer-type FAI was defined by an LCEA of ≥40° (Figure 4) [16,17,18,19]. For CT-MIP evaluation, the maximal cross-sectional area of the femoral head was initially measured on axial CT images using a circular region of interest (ROI). MIP reformatted images with a slab thickness of 10 mm were subsequently generated to enable a comprehensive single-slice evaluation of the acetabular roof. The acetabular coverage area was calculated by manual freehand delineation of the acetabular roof margins covering the femoral head, while preserving the femoral head contours. The ACI was defined as the ratio of the acetabular coverage area to the area of the femoral head (Figure 5).

CT-MIP reconstructions were used to facilitate a global and reproducible evaluation of acetabular coverage. The rationale for using CT-MIP was to integrate information derived from multiple axial slices into a single en-face representation of the acetabular roof, thereby reducing slice-selection bias and enabling standardized quantitative assessment independent of patient positioning. Compared with conventional two-dimensional CT measurements, CT-MIP provides a holistic visualization of acetabular overcoverage and allows calculation using routinely available workstation tools without the need for dedicated software.

### 2.3. Statistical Analysis

Statistical analyses were performed using SPSS Statistics version 26.0 (IBM Corp., Armonk, NY, USA). The distribution of continuous variables was assessed using the Shapiro–Wilk test. Associations between the presence of bone marrow edema and the presence of FAI, FAI subtypes, and acetabular overcoverage subtypes were evaluated using the chi-square test. Continuous variables showing a normal distribution were expressed as mean ± standard deviation and compared between groups using the student *t*-test. To address the potential non-independence of bilateral hip data, additional patient-level and within-patient paired analyses were performed, including paired *t*-tests and McNemar tests, as appropriate. The diagnostic performance of angular measurements was assessed using receiver operating characteristic (ROC) curve analysis. Statistical differences between ROC curves were evaluated using the DeLong test with MedCalc software (version 23.4.3; MedCalc Software Ltd., Ostend, Belgium). Intraobserver and interobserver agreement were assessed by calculating the intraclass correlation coefficient (ICC). A *p* value of <0.05 was considered statistically significant.

## 3. Results

The mean ages of the case and control groups were 49.73 ± 15.80 years and 49.77 ± 15.09 years, respectively. In the case group, 14 patients (53.8%) were female and 12 (46.2%) were male. Focal atypical bone marrow edema was identified in the right hip in 11 patients, the left hip in 11 patients, and bilaterally in 4 patients. The majority of patients with bone marrow edema on MRI (82.7%) demonstrated FAI, whereas FAI was present in only 34.6% of subjects without bone marrow edema. This finding indicates a strong association between atypical femoral head bone marrow edema and the presence of FAI (*p* < 0.001). When FAI subtypes were compared, the prevalence of bone marrow edema on MRI was significantly higher in hips with pincer-type morphology than in other subtypes (*p* < 0.001; Table 1). Acetabular overcoverage subtypes are detailed in Table 2. Although global acetabular overcoverage constituted the majority of cases, no statistically significant difference was observed between groups (*p* > 0.05). Regarding the localization of bone marrow edema, 66.7% of lesions were located laterally, with the most common distribution being posterolateral (43.3%). Similarly, posterolateral edema was observed in 44.4% of patients with FAI and in half of the patients with acetabular overcoverage. However, no statistically significant association was identified between edema location and impingement subgroups (*p* > 0.05). The mean size of bone marrow edema was 14.07 ± 4.02 mm, and no significant association was found between edema size and the presence of impingement (*p* > 0.05). The lack of significant associations between bone marrow edema location, edema size, and FAI subtypes may reflect limited statistical power related to the small sample size and heterogeneity of edema patterns.

**Figure 6:**
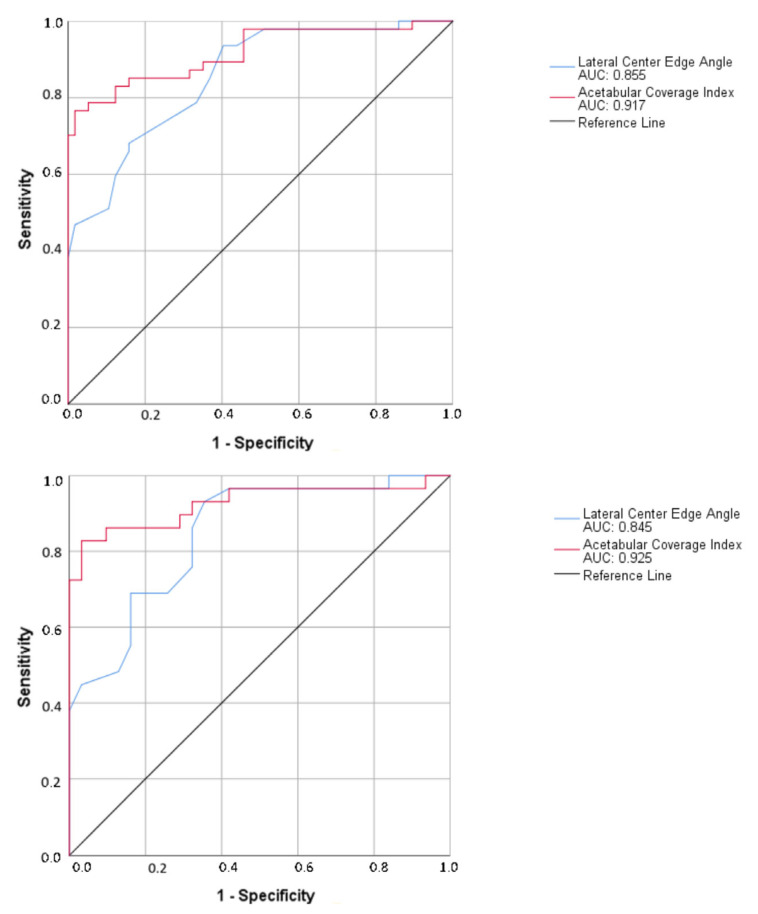
ROC curves showing the performance of LCEA and ACI for identifying pincer-type femoroacetabular impingement. The upper panel includes all evaluated hip joints, whereas the lower panel is restricted to hips with bone marrow edema and matched control hips.

The mean alpha angles were 49.64 ± 13.90 for the case group and 43.05 ± 10.43 for the control group, demonstrating a statistically significant difference (*p* = 0.007). The average LCEA values were 36.81 ± 9.38 for the case group and 32.06 ± 6.55 for the control group (*p* = 0.004). The average ACI in the case group was 0.92 ± 0.08, while in the control group, it was 0.87 ± 0.06 (*p* = 0.003). In ROC analysis for identifying pincer-type FAI, ACI showed remarkable diagnostic performance, with an area under the curve (AUC) of 0.917 (95% confidence interval [7], 0.847–0.962). LCEA, on the other hand, exhibited a lower AUC of 0.855 (95% CI, 0.772–0.916) (Figure 6). The DeLong test showed that ACI had a substantially higher AUC than LCEA (ΔAUC = 0.062; 95% CI, 0.026–0.062; *p* = 0.017). An ACI cutoff value of 0.93 yielded significant diagnostic differentiation through ROC analysis, with a sensitivity of 78.7%, specificity of 93%, and overall accuracy of 85%.

In the hip-based analysis, the distribution of FAI and its subtypes between hips with bone marrow edema and age- and sex-matched ipsilateral control hips is presented in Table 3. Hips with pincer-type FAI demonstrated significantly higher LCEA values than hips without pincer-type FAI (39.8 ± 8.1 and 29.3 ± 6.1, respectively; *p* < 0.001). Similarly, ACI values were significantly higher in the presence of pincer-type FAI (0.951 ± 0.056 and 0.848 ± 0.051, respectively; *p* < 0.001). At the individual hip level, both acetabular overcoverage parameters demonstrated robust diagnostic performance for identifying pincer-type FAI. ACI achieved an AUC of 0.925 (95% CI, 0.851–1.000), while LCEA showed an AUC of 0.845 (95% CI, 0.747–0.943) (Figure 6). Overall, these findings indicate that both ACI and LCEA reliably reflect acetabular overcoverage associated with pincer-type FAI, with consistent performance across patient-based and hip-based analytical approaches.

**Figure 7:**
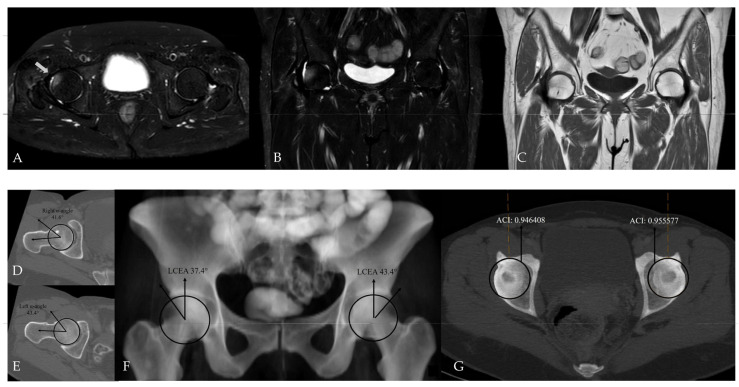
Hip MRI demonstrates focal bone marrow edema in the anterolateral aspect of the right femoral head on (**A**) axial (white arrow) and (**B**) coronal fat-suppressed T2-weighted images. (**C**) Coronal T1-weighted image shows no underlying structural abnormality. (**D**,**E**) CT demonstrates normal alpha angle measurements. (**F**) LCEA measurements indicate pincer-type femoroacetabular impingement only on the left side. (**G**) However, using an ACI cut-off value of 0.93, bilateral pincer-type femoroacetabular impingement can be demonstrated.

**Figure 8:**
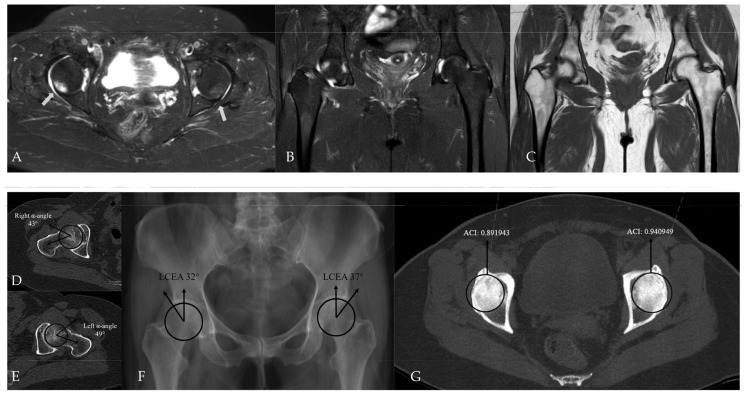
Hip MRI demonstrates bilateral posterolateral femoral head bone marrow edema on (**A**) axial (white arrows) and (**B**) coronal fat-suppressed T2-weighted images, more pronounced on the right, with associated right hip joint effusion. (**C**) Coronal T1-weighted image shows no structural abnormality. (**D**,**E**) CT shows normal alpha angle measurements, and (**F**) LCEA values are within normal limits. (**G**) However, the ACI exceeds the 0.93 cut-off value on the left, while CT–MIP images demonstrate posterior acetabular rim overcoverage on the right without marked posterior wall lateralization.

**Figure 9:**
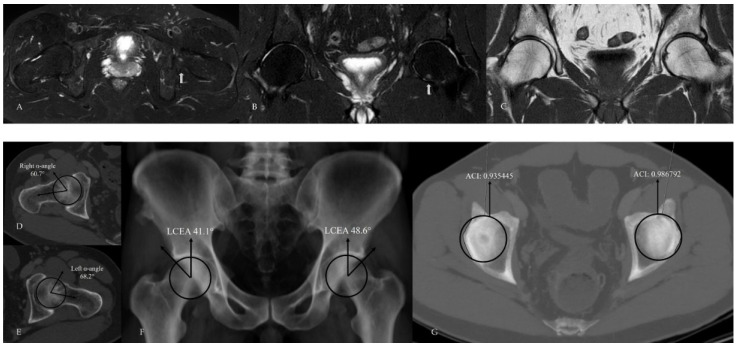
Hip MRI demonstrates focal bone marrow edema in the inferior aspect of the left femoral head on (**A**) axial and (**B**) coronal fat-suppressed T2-weighted images (white arrow). (**C**) Coronal T1-weighted image shows no associated additional imaging findings. (**D**,**E**) CT demonstrates alpha angle measurements, and (**F**) LCEA measurements indicate bilateral mixed-type femoroacetabular impingement. (**G**) Acetabular overcoverage is confirmed using the ACI.

In within-patient paired analyses, no statistically significant differences were observed between the hip with bone marrow edema (BME) and the contralateral hip in terms of acetabular coverage measurements. LCEA values did not differ significantly between the BME side and the contralateral side (36.9 ± 10.6 and 35.8 ± 9.2, respectively; paired *t*-test, *p* = 0.312). Likewise, ACI values showed no significant difference between sides (0.917 ± 0.085 and 0.903 ± 0.092, respectively; paired *t*-test, *p* = 0.264). Notably, strong positive correlations were observed between sides for both LCEA (r = 0.895) and ACI (r = 0.804), with both correlations reaching statistical significance (*p* < 0.001), indicating a high degree of bilateral acetabular morphological similarity. After exclusion of cases with bilateral bone marrow edema, the results of the paired McNemar analysis comparing the presence of pincer-type FAI between the BME side and the contralateral hip are presented in Table 4. These additional within-patient analyses were performed to explicitly address the potential non-independence of bilateral hip data (Figure 7, Figure 8 and Figure 9).

Intraobserver and interobserver reliability for ACI measurements was excellent (Table 5). Alpha angle and LCEA measurements also demonstrated excellent interobserver reliability. These findings indicate consistently high reproducibility across observers. No systematic differences were identified between repeated measurements.

## 4. Discussion

The findings of the present study suggest that atypical femoral head bone marrow edema of unknown etiology may represent an MRI manifestation of early mechanical stress related to FAI, particularly pincer-type overcoverage. Unlike the prevailing literature, in which femoral head bone marrow edema is predominantly regarded as a secondary phenomenon arising from identifiable primary pathologies, our results highlight its potential role as a functionally relevant MRI finding associated with pincer-type FAI. Notably, FAI was identified in 82.7% of the cases included in our cohort who exhibited atypical femoral head bone marrow edema. Viewed in this light, the present study provides the first quantitative validation of a clinically meaningful imaging observation that has long been recognized by experienced radiologists but has remained largely confined to expert impression in the absence of objective supporting evidence.

FAI is characterized by abnormal mechanical loading resulting from aberrant contact between the femoral head–neck junction and the acetabulum during hip joint motion. Repetitive mechanical stress induces microtrauma within the subchondral bone, leading to the development of histologically subtle fissures. This process triggers stromal edema and a fibrovascular reparative response, which constitute the histopathological correlate of bone marrow edema observed on MRI and represent the initial step in the cascade toward osteoarthritis, characterized by the coexistence of cyst formation and sclerosis [20,21]. Several decades ago, the development of synovial pits and subchondral cysts—described on conventional radiographs as early manifestations of impingement—was similarly interpreted within a comparable spectrum of osseous responses, despite differences in their anatomical and histological origins [20,21,22]. However, advances in imaging technology now allow these alterations to be detected by MRI well before they become apparent on plain radiographs. Bone marrow edema, which is not detectable on conventional radiography but is readily visualized on MRI, may represent an early-detected imaging manifestation within the disease spectrum, without implying temporal precedence. Accordingly, bone marrow edema that may initially appear innocuous should be regarded as a potentially relevant imaging finding within the disease continuum and interpreted as an alerting sign of underlying FAI.

Beyond the mere presence of bone marrow edema, its anatomic location may also serve as an alerting feature with respect to the FAI subtype and its clinical relevance. In our study, the prevalence of atypical focal bone marrow edema was significantly higher in pincer-type FAI than in cam-type FAI (*p* = 0.000, Table 2). The majority of bone marrow edema lesions (66.7%) were laterally located, a finding that may be attributed to the high degree of acetabular overcoverage observed in our study population. Indeed, lateral overcoverage based on coxa profunda, which represents an inherent component of pincer-type FAI, leads to a lateral shift in mechanical loading and results in increased traumatic stress on the lateral subchondral region through a pincer-like mechanism involving the femoral head–neck junction. The literature further suggests that regions exposed to the greatest mechanical stress may correspond either to sites of direct contact or to areas affected by compensatory mechanisms, including a contrecoup pattern of injury [9,11]. In addition, prior studies by Ganz et al. and Leunig et al. have indicated that increased acetabular coverage is associated with femoral head bone marrow edema through marginal chondral contact loading, elevated subchondral stress, and microvascular alterations during osteoarthritis progression [9,23]. Although the pathogenesis of labral and chondral damage in FAI has been extensively investigated, data examining its association with femoral head bone marrow edema remain limited, and the existing literature does not provide a clear explanation for the imaging findings observed in this patient group.

Another important strength of our study lies in the evaluation of FAI in these cases using a practical CT-MIP approach, in addition to routinely employed diagnostic measurements, with the technical details of this method described in detail in Section 2. Indeed, ongoing technological innovation and advancement—both in medicine and across all aspects of daily life—have generated an increasing demand for imaging techniques that enable more detailed, reliable, and simultaneously practical assessment. In this regard, we evaluated ACI measurements rapidly and effectively using CT-MIP, a technique available on nearly all standard workstations and applicable without the need for additional software or added cost. The high intraobserver and interobserver agreement observed in our measurements (0.937–0.965) represents a key indicator of the technique’s ease of use and reproducibility in routine clinical practice. Using a similar approach, Dandachli previously performed evaluations focusing on pelvic tilt and acetabular orientation [24]. Furthermore, a review by Ghaffari et al. suggests that comparable methods have been employed for subtyping acetabular overcoverage [15]. Notably, Nardi et al. reported that 3D imaging-based measurements provide higher diagnostic accuracy than two-dimensional approaches for the classification of FAI subtypes and demonstrate better concordance with surgical assessment [12]. Undoubtedly, this advantage primarily stems from the susceptibility of conventional radiographic methods to even minimal variations in pelvic tilt and rotation, as they are inherently based on two-dimensional projections. In comparison, CT- and MRI-based 3D models offer substantially more detailed and reliable information regarding acetabular rim configuration, the degree of coverage, and femoroacetabular relationships, thereby enabling a more comprehensive evaluation [10,17,25,26,27]. Moreover, driven by rapid technological advances, there has been an increasing tendency—beyond the emphasis on early diagnosis as a fundamental goal of contemporary healthcare—toward more detailed planning, particularly in the preoperative setting [28]. To this end, dynamic simulations are performed using 3D software, or classical measurements, such as the lateral center-edge angle, are redefined on 3D imaging datasets to improve diagnostic accuracy [23,29,30]. From this perspective, we believe that ACI measurements performed on MIP images may also contribute to the early detection of FAI. In routine clinical practice, the hip joint is often relegated to a secondary consideration on lower abdominal or pelvic CT examinations performed for a variety of clinical indications. Incorporating the hip into routine assessment through MIP-based ACI measurement may, nevertheless, play a role in the early diagnosis of FAI, particularly in young adults who represent the primary target population within the healthy aging paradigm. Given the simplicity of the method, particularly when combined with artificial intelligence–assisted automated analyses, its implementation is likely to become faster and more widespread in the near future.

In our study, CT–MIP based ACI measurements demonstrated superior diagnostic performance for detecting pincer-type FAI compared with LCEA measurements, with significantly higher AUC values. In line with the study by Chadayammuri et al., LCEA measurements in our CT-based virtual pelvis radiograph models were obtained using the most lateral acetabular rim as the reference point [31]. Conventional two-dimensional angular measurements demonstrated high diagnostic performance for the diagnosis of FAI, consistent with the existing literature, while yielding an FAI prevalence in the control group that was comparable to previously reported rates [32,33]. In a study by Werner et al. investigating normative values, conventional radiographic measurements yielded a mean LCEA of 33.6°, whereas in our study, mean LCEA values within a similar range were observed in the control group (32.06°) [34]. Falgout et al. reported that LCEA measurements obtained from CT tend to be higher than those derived from conventional methods, attributing this difference to discrepancies between weight-bearing pelvic radiographs and supine CT examinations [30]. Similarly, Figueroa et al. noted that LCEA values derived from CT-based virtual pelvis models may be higher than those obtained from plain radiographs; however, they emphasized that this difference is unlikely to be clinically meaningful [35]. Despite differences in measurement standardization across these studies, the reported values are consistent with the mean values observed in our control group, suggesting that, although the sample size is limited, our control cohort represents a reliable reference population.

It should be acknowledged that the ROC analyses evaluating ACI and LCEA for identifying pincer-type FAI do not represent independent diagnostic tests in the strict sense, as acetabular overcoverage is a defining morphological feature of pincer-type FAI. Accordingly, the high AUC values observed for ACI are expected by design and should be interpreted as reflecting the discriminatory capacity of quantitative acetabular coverage parameters, rather than implying clinical diagnostic superiority. In this context, the ROC analyses are intended to illustrate the relative performance of different morphologic metrics in characterizing acetabular overcoverage, rather than to validate ACI as an independent diagnostic tool.

Given the potential non-independence of bilateral hip measurements, we conducted additional patient-level and within-patient paired analyses to mitigate the risk of inflated statistical significance. The absence of significant differences in acetabular coverage parameters between the BME side and the contralateral hip, together with the strong bilateral correlations observed, suggests a high degree of intrinsic symmetry in acetabular morphology. These findings support the robustness of the main results and indicate that the observed associations between acetabular overcoverage parameters and pincer-type FAI are not driven by within-patient clustering effects. Overall, acetabular overcoverage and pincer-type morphology appear to represent largely bilateral structural characteristics rather than focal unilateral abnormalities. In this context, the unilateral manifestation of bone marrow edema may reflect asymmetric mechanical loading or localized stress acting on an otherwise bilaterally similar acetabular morphology, rather than intrinsic side-specific differences in acetabular structure. Therefore, the observed association between acetabular overcoverage parameters and pincer-type FAI cannot be attributed to artificial inflation resulting from bilateral inclusion of hips.

Although previous studies have investigated FAI morphology using three-dimensional CT- or MRI-based techniques, the present study differs in its clinical focus and analytical perspective. Rather than proposing a novel morphologic parameter, we specifically focused on the underexplored imaging scenario of focal femoral head bone marrow edema of unknown etiology and examined its association with acetabular overcoverage, particularly pincer-type FAI. Importantly, our approach emphasizes the use of CT-MIP images to derive acetabular coverage measurements with standard workstation tools, highlighting a practical and reproducible method readily applicable in routine clinical settings. In this context, the novelty of the study lies not in the introduction of new imaging technology, but in the integration of established 3D assessment techniques with a clinically challenging and previously under-characterized MRI finding.

This study has several limitations. First, the relatively small sample size limited the generalizability of the findings, the applicability of three-dimensional measurement techniques, and the precision of the proposed cutoff values; therefore, the results should be regarded as preliminary. In addition, the limited sample size may have reduced statistical power to detect associations between bone marrow edema characteristics, such as location and size, and specific FAI subtypes, and thus these negative findings should be interpreted cautiously. The use of CT imaging represents an inherent limitation due to exposure to ionizing radiation and the potential for selection bias, as not all patients with hip pain routinely undergo CT examination. However, no additional CT scans were acquired for research purposes, and all analyses were performed exclusively on pre-existing CT examinations obtained for routine clinical indications. Furthermore, although alternative causes of bone marrow edema were systematically excluded based on imaging findings and available clinical information, standardized clinical, laboratory, or longitudinal imaging follow-up was not available for all patients, which may have introduced a degree of misclassification bias. Another limitation is the long inclusion period from 2007 to 2025, during which imaging hardware, acquisition protocols, and diagnostic concepts related to FAI evolved. To mitigate potential temporal bias, all measurements were performed retrospectively using standardized criteria applied consistently to archived datasets across the entire study period. In addition, the ROC analyses should be interpreted with caution, as the evaluated parameters directly quantify acetabular coverage, which is a defining morphological feature of pincer-type FAI. Methodologically, the manual contour-based three-dimensional measurement approach demonstrated good interobserver agreement but requires a substantial learning curve. Moreover, the applied three-dimensional technique did not allow assessment of femoral version or torsion, and the effects of pelvic tilt and rotation could not be fully eliminated. The use of static imaging also precluded evaluation of dynamic impingement mechanics. Finally, the absence of direct correlations between imaging findings and symptom severity, functional scores, or clinical tests, as well as the lack of volumetric or intensity-based quantitative assessment of bone marrow edema, further limits interpretation of the clinical significance of the observed imaging findings. Due to the retrospective and cross-sectional study design, no conclusions can be drawn regarding temporal precedence, prognostic value, or predictive relevance for disease progression or clinical outcomes.

## 5. Conclusions

Atypical femoral head bone marrow edema of unknown etiology may be regarded as an important radiologic finding suggestive of underlying pincer-type FAI, while CT–MIP-based ACI measurements demonstrate promising diagnostic performance for the detection of acetabular overcoverage. However, confirmation of these findings in larger cohorts and multicenter studies will enhance the generalizability of the results.

## Figures and Tables

**Table 1:** FAI types categorized by bone marrow edema status on MRI.

FAI Type		MRI (-), n (%)	MRI (+), n (%)	Total, n (%)
FAI (-)		34 (65.4)	9 (17.3)	44 (41.3)
FAI (+)	Cam type	6 (11.5)	8 (15.4)	13 (13.5)
	Pincer type	10 (19.2)	29 (55.8)	39 (37.5)
	Mixed type	2 (3.8)	6 (11.5)	8 (7.7)
Total		52 (100)	52 (100)	104 (100)

**Table 2:** Distribution of MR Abnormalities Across Pincer-Type FAI Subtypes.

Pincer Type FAI Subtype	MRI (-), n (%)	MRI (+), n (%)	Total, n (%)
Global overcoverage	7 (58.3)	24 (68.6)	31 (66)
Focal anterior overcoverage	2 (16.7)	0	2 (4.2)
Acetabular retroversion	1 (8.3)	5 (14.3)	6 (12.8)
Focal posterior overcoverage	2 (16.7)	6 (17.1)	8 (17.0)
Total	12 (100)	35 (100)	47 (100)

**Table 3:** Hip-based distribution of femoroacetabular impingement subtypes in hips with and without bone marrow edema.

Focal Femoral Bone Marrow Edema	(-) n (%)	Femoroacetabular Impingement				Total n (%)
		(-) n (%)	(+) n (%)			30 (%50)
		21 (%70)	9 (%30)			
			Femoroacetabular Impingement Type			
			Cam	Pincer	Mixt	
			1 (%3.3)	6 (%20)	2 (%6.7)	
	(+) n (%)	Femoroacetabular Impingement				Total n (%)
		(-) n (%)	(+) n (%)			30 (%50)
		3 (%10)	27 (%90)			
			Femoroacetabular Impingement Type			
			Cam	Pincer	Mixt	
			6 (%20)	17 (%56.7)	4 (%13.3)	
Total n (%)		24 (%40)	36 (%60)			60 (%100)

**Table 4:** Paired analysis of pincer-type femoroacetabular impingement in bone marrow edema and contralateral hips.

Variable	Contralateral Pincer Type FAI		Contralateral Pincer Type FAI
	(-) n (%)	(-) n (%)	
BME side pincer type FAI (-) n (%)	6 (%75)	2 (%25)	8 (%100)
BME side pincer type FAI (+) n (%)	2 (%14.3)	12 (%85.7)	14 (%100)
Total n (%)	8 (%36.4)	14 (%63.6)	22 (%100)

BME: Bone marrow edema, FAI: Femoroacetabuler impingement.

**Table 5:** Inter-observer and intra-observer reliability analysis for measured variables.

Variable Type	Observers	Coefficient Value	95% CI	*p* Value
Alfa angle	Observer 1 vs. Observer 2 (ICC)	0.968	0.947–0.980	0.000
LCEA	Observer 1 vs. Observer 2 (ICC)	0.963	0.945–0.975	0.000
ACI	Observer 1 vs. Observer 2 (ICC)	0.937	0.907–0.958	0.000
ACI	Observer 1 first vs. second time (ICC)	0.965	0.948–0.976	0.000

LCEA: Lateral center edge angle, ACI: acetabular coverage index, ICC: Intraclass correlation coefficient, CI: Confidence Interval.

## Data Availability

The data presented in this study are available on request from the corresponding author due to ethical restrictions related to patient privacy.

## Abbreviations

ACI	Acetabular coverage index
AUC	Area under the curve
CI	Confidence interval
CT	Computed tomography
CT-MIP	Computed tomography maximum intensity projection
FAI	Femoroacetabular impingement
FOV	Field of view
FS	Fat-suppressed
ICC	Intraclass correlation coefficient
LCEA	Lateral center–edge angle
MIP	Maximum intensity projection
MRI	Magnetic resonance imaging
MR	Magnetic resonance
NEX	Number of excitations
OA	Osteoarthritis
PACS	Picture archiving and communication system
PD	Proton density
ROC	Receiver operating characteristic
ROI	Region of interest
TE	Echo time
TR	Repetition time

## Appendix A

Detailed MRI parameters for all pulse sequences acquired in the study, as presented in the table below, are provided in Appendix A.

**Table A1:** MRI pulse sequence parameters.

Imaging Parameters	PD FS Coronal	PD FS Axial	T1W Coronal	T2W FS Coronal	T2W FS Axial
TR (ms)	3282	4376	575	4198	4169
TE (ms)	30	30	10	80	80
NEX	1.5	1.5	1.5	1.5	1.5
Flip Angle (deg)	90	90	90	90	90
Slice thickness (mm)	5	5	5	5	5
Interslice gap (mm)	1	1.5	1	1	1.5
Matrix	232 × 238	216 × 241	272 × 264	240 × 220	272 × 249
FOV (mm)	300	300	300	300	300

FOV, field of view; FS, fat-suppressed; NEX, number of excitations; TE, echo time; TR, repetition time; Deg, degrees; ms, milliseconds; mm, milimeters.
